# A comprehensive evaluation of lightweight deep learning models for tomato disease classification on edge computing environments

**DOI:** 10.1038/s41598-026-42439-6

**Published:** 2026-03-05

**Authors:** Trong-Minh Hoang, Van-Hau Bui, Van-Son Nguyen, Duc-Thang Doan, Hoang-Anh Dang, Anh-Thu Pham

**Affiliations:** 1 Posts and Telecommunications Institute of Technology, Hanoi, Vietnam; 2 University of Economics-Technology for Industries, Hanoi, Vietnam; 3 Hanoi Open University, Hanoi, Vietnam

**Keywords:** Edge deployment, Explainable AI, Grad-CAM, Hybrid vision transformer, Lightweight deep learning, MobilePlantViT, Model benchmarking, Plant disease detection, PlantVillage dataset, Precision agriculture, Tomato leaf disease, Computational biology and bioinformatics, Engineering, Mathematics and computing, Plant sciences

## Abstract

To achieve agricultural automation, deep learning applications for early and accurate disease detection in tomato plants have been extensively developed. However, there is a fundamental trade-off between computational efficiency and diagnostic accuracy in resource-constrained agricultural edge environments. This paper proposes an evaluation framework for seven architectures that represent standard, efficient, and hybrid CNN structures to assess their implementation potential. Through evaluations of explainability, computational efficiency, and diagnostic performance, seven lightweight architectures (ShuffleNetV2, MobileNetV3-Small, SqueezeNet, MobilePlantViT, DenseNet121, ResNet50, and VGG16) are thoroughly examined. Three significant findings are derived from experiments conducted on a subset of tomato diseases in the PlantVillage dataset. First, the MobilePlantViT architecture accurately strikes the ideal balance between efficiency and performance. Second, in order to quantitatively assess the explainability of XAI models (Grad-CAM, SHAP, and LIME) and identify the best option for edge devices, we propose the perturbation stability score (PSS) metric. Third, we test CPU inference measurements to better reflect the actual scenario and find that the hybrid design effectively leverages parallel computing. According to these findings, MobilePlantViT is the ideal architecture for applications that require operation on edge devices with limited resources and achieve high diagnosis accuracy (above 99.5%).

## Introduction

Plant diseases are a major concern for agricultural production, as yield losses can exceed 30% globally^1^. Among high-value crops, the tomato, widely grown worldwide, is affected by various diseases due to geographic and climatic variations. Most of these diseases are manifested by obvious symptoms on leaves, stems, and fruits^2^ and require detection. In traditional farming, experienced farmers can identify diseases and make adaptive decisions. However, with the help of current IoT systems and visual science methods, symptom identification is becoming more accurate, stable, and efficient, which will lead to its widespread use in smart agriculture^3^.

In recent years, visual recognition solutions have made significant progress, shifting the paradigm for automated plant disease diagnosis. In particular, convolutional neural networks (CNNs) have demonstrated superior performance on collected datasets, with classification accuracy exceeding 98% under controlled laboratory conditions (PlantVillage)^4^. However, in addition to real-world data samples that can lead to discrepancies in accuracy, the resource constraints of practical devices pose a significant obstacle to deployment when high-performance ML models require substantial computational resources. In particular, to combine authentic farmer judgment with diagnostic model results, model explainability becomes an important factor in machine learning solutions^5^. Therefore, feasible solutions that meet the requirements of edge deployment and explainability are among the challenges that need to be addressed^6^. In practical agricultural scenarios, disease diagnosis systems are increasingly deployed on edge devices such as embedded IoT cameras, mobile phones, and handheld scanners, where GPU acceleration is unavailable and computational resources are strictly limited^7^. Therefore, evaluating lightweight models under CPU-only, single-thread inference settings, along with explainable decision mechanisms, is essential to assess their feasibility in real-world edge computing environments.

In this approach, lightweight machine learning architectures have been proposed, such as MobileNetV3 ^8^, ShuffleNet ^9^ and SqueezeNet ^10^, which significantly reduce the model size and latency while maintaining competitive accuracy. These compact CNNs are applied in real-time, resource-constrained scenarios for tomato disease detection^2^, enabling the ability to explain results and interpret the decision^6^.

More recent works have further advanced this direction. The study in ^11^ introduced LDL-MobileNetV3S, an enhanced lightweight MobileNetV3-Small model for potato leaf disease diagnosis, achieving 94.9% accuracy with only 1.5 M parameters and demonstrating strong potential for edge deployment. Similarly, an improved ShuffleNetV2 architecture was proposed in ^12^, achieving 96.7% accuracy on field-crop leaf datasets while reducing latency and memory usage. Comparative analyses have also been conducted across different lightweight CNNs; for example, ^13^ evaluated several compact architectures (MobileNetV3, EfficientNet-B0, and ShuffleNetV2) for wheat rust classification and found that MobileNetV3 offers the optimal trade-off between accuracy and efficiency. Additionally, AgriFusionNet^14^ is a lightweight fusion model that combines features from MobileNet and ShuffleNet, yielding competitive results on crop disease datasets.

Recently, the Vision Transformer (ViT)^15^ has revolutionised computer vision by demonstrating that attention-based architectures can surpass CNNs on large datasets when adequately pre-trained. This advancement has led to various efficient and mobile-optimised variants. MobileViT^16^ represents a significant development, introducing a hybrid architecture that combines convolutional layers for local feature extraction with transformer blocks for global context, thereby achieving better trade-offs between accuracy and efficiency for mobile vision tasks compared to pure CNNs or standard ViTs. Following this, recent studies have examined hybrid architectures for agricultural purposes. The authors in^17^ proposed a lightweight CNN-Transformer hybrid for rice disease classification, achieving 96.7% accuracy with 3.2 million parameters. However, their model’s lack of evaluation on the PlantVillage benchmark restricts its comparability with existing research. In^18^, a Swin Transformer variant was applied to multi-crop disease detection, demonstrating strong performance (98.1% accuracy) but with high computational cost (22M parameters, 4.5G FLOPs), making it impractical for edge deployment.^19^ proposed an improved MobileViT for tomato disease recognition on the Ai Challenger dataset, incorporating Squeeze-and-Excitation blocks, Global Attention Mechanism (GAM), and Mish activation to boost accuracy to 88.86%. While this work demonstrates the potential of MobileViT variants in agricultural vision, it does not evaluate the baseline MobileViT architecture on the widely adopted PlantVillage benchmark, nor does it compare against established lightweight CNNs under standardised conditions. Crucially, their approach lacks any form of model interpretability analysis, leaving the trustworthiness of predictions unverified. Similarly,^20^ applied MobileViT to apple disease detection using a proprietary orchard dataset, achieving 93.2% accuracy. However, ViT-based approaches lack comparison with lightweight CNNs, and explainability is not yet a concern for these models. For instance, a recent CNN-Transformer hybrid architecture^21^ effectively integrates local feature extraction and global contextual modelling to improve the classification of soybean leaf diseases, demonstrating superior performance over conventional CNN-based approaches. Similarly, an enhanced deep learning framework proposed in^22^ focuses on architectural optimization and robust feature representation for crop disease identification, demonstrating the versatility of advanced deep learning models in complex agricultural imaging scenarios. Unlike existing transformer-based approaches^21,22^, our study emphasizes edge-oriented deployment under CPU-only constraints.

Along with the development of machine learning techniques, explainable AI (XAI) techniques have been developed to address the transparency of decisions. In the agricultural domain, gradient-based visualization methods, such as Grad-CAM ^23^, remain the most widely applied method for highlighting disease-discriminating regions. In  ^24^, a CNN-based cassava disease classification using Grad-CAM was used to confirm symptomatic leaf regions in 87% of test cases. However, this method tends to be qualitative in its analysis and lacks a quantitative assessment of the consistency of the explanation. To further enhance interpretability and reliability,^6^ proposed a lightweight yet explainable CNN model that integrates Grad-CAM and LIME visualizations, achieving robust interpretability across multiple crop datasets while maintaining edge device efficiency. In^25^, Grad-CAM, LIME, and SHAP were compared on wheat disease datasets, finding that Grad-CAM provides superior visual understanding, while SHAP provides more detailed properties. However, a detailed comparison of XAI with lightweight edge models is not fully addressed in these studies.

Furthermore, benchmark comparisons in agricultural AI remain fragmented. Most existing evaluations focus only on conventional CNNs, ignore lightweight or hybrid transformer architectures, and lack XAIs. Therefore, there is still no unified quantitative comparison that integrates performance, efficiency, and explainability across CNNs, efficient CNNs, and hybrid transformer models. This research gap is particularly significant for making informed decisions about model selection in practical agricultural applications. To focus on this goal, we present in this paper a comprehensive comparison of lightweight architectures for tomato disease diagnosis, their explainability, and their potential for deployment on the edge with the following key contributions:

1. Performance comparison of seven representative lightweight model architectures in categories such as standard CNNs (VGG16, ResNet50, DenseNet121), efficient CNNs (MobileNetV3-Small, ShuffleNet, SqueezeNet), and hybrid convolutions (MobilePlantViT based on MobileViT^16^).
2. Quantitative explainability evaluation over three XAI methods (Grad-CAM, LIME, SHAP) with a novel Perturbation Stability Score (PSS) metric for the best selection of consistently stable explanations across all architectures.
3. Comprehensive edge deployment feasibility analysis through CPU-based inference latency measurements under both optimal (4-thread) and constrained (1-thread) configurations, demonstrating real-time capability for agricultural applications.

## Related work

Deep learning has become the key approach for automated plant disease diagnosis since the introduction of the PlantVillage dataset ^26^, which enabled early CNN models, such as those models in  ^27^ and  ^28^, to achieve accuracies above 99% under laboratory conditions. However, they cannot adapt to the real environments, mainly due to lighting variation, background complexity, and image noise ^29,30^. These need additional preprocessing before being trained with machine learning models. Otherwise, lightweight ML architectures must be considered for deployment on edge devices.

ShuffleNet ^9^ and SqueezeNet ^10^ employ efficient depthwise convolutions and channel shuffling to minimise parameters and latency while preserving reasonable accuracy with minimal computational complexity. Similarly, the LDL-MobileNetV3S model proposed by the authors in^11^ attained 94.9% accuracy using only 1.5 M parameters. Beyond CNN models, the hybridisation of convolutional and transformer architectures has developed recently to enhance the accuracy and reduce the complexity. The MobileViT framework ^16^ and its variants ^17,19^ demonstrate that its hybrid approach can bring good performance results compared to conventional Vision Transformers (ViT) ^15^. Hence, this approach can be suitable for low-resource environments as edge computing.

In recent years, explainable AI (XAI) has been used in plant disease fields to enhance transparency and trust. Grad-CAM ^23^ used a gradient-based visual method for highlighting disease-discriminative regions. The authors in^24^ used CAM to recognize cassava disease. The authors in^6^ introduced a lightweight and explainable CNN that integrates Grad-CAM and LIME for achieving interpretable and resource-efficient diagnosis across various crop datasets. Moreover, the authors in^25^ compared Grad-CAM, LIME, and SHAP for wheat disease classification. In fact, while emerging high-performance CNNs and hybrid transformers have improved accuracy and efficiency, comparative analyses that unify performance, computational cost, and explainability remain underexplored. Therefore, this study proposes a comparative analysis of lightweight CNN and hybrid transformer models for tomato disease diagnosis, combining quantitative evaluation of multiple XAI techniques to evaluate their potential deployment on agricultural edge devices in practice. Moreover, YOLO variants (YOLOv9–YOLOv13 and YOLO26) offer improved accuracy–efficiency trade-offs and real-time suitability, while transformer-based detectors such as RF-DETR have demonstrated superior detection performance with competitive inference speed^31–33^. These models will be benchmarked for practical deployment in complex field environments.

## Methodology

**Fig. 1:**
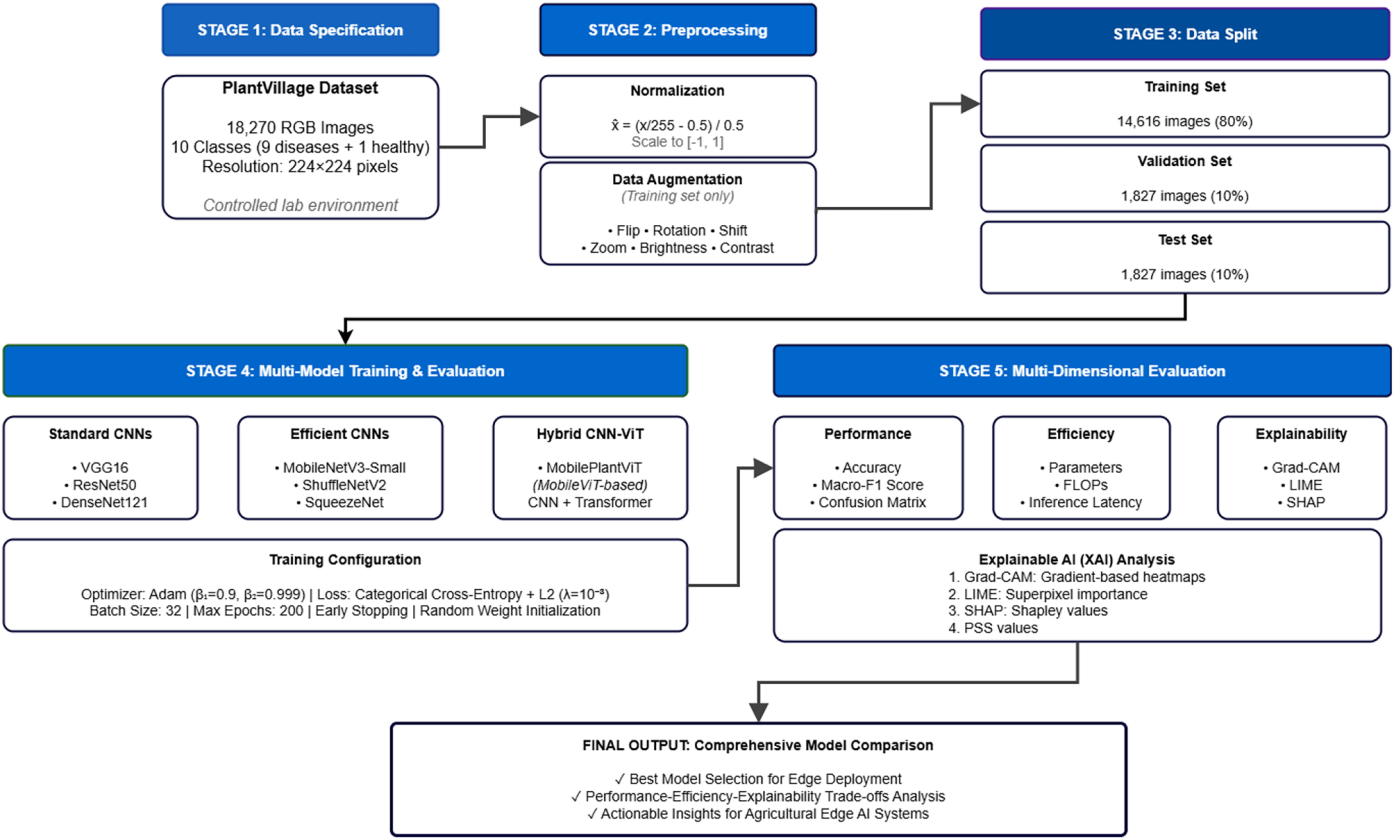
The pipeline of the tomato disease diagnosis framework.

In order to perform the comparison effectively, we propose an interpretable tomato disease diagnosis framework consisting of four sequential stages: (i) data specification and preprocessing, (ii) data partitioning, (iii) multi-model training and performance evaluation, and (iv) explainable AI (XAI) analysis. The main pipeline is illustrated in Figure 1.

### Dataset specification

We utilize the tomato subset of the PlantVillage dataset^26^, which contains 18,270 RGB leaf images across 10 classes: There are nine diseases (bacterial spot, early blight, late blight, leaf mold, Septoria leaf spot, two-spotted spider mite, target spot, tomato yellow leaf curl virus, and tomato mosaic virus) and one healthy class. The dataset has a medium class imbalance, with class sizes ranging from 1,300 to 2,152 images. All images are taken in a controlled laboratory environment with uniform backgrounds. Before preprocessing, all images are resized to $224 \times 224$ pixels, matching the standard input resolution.

### Data preprocessing

Let $\mathscr {D} = \{({\bf x}_i, y_i)\}_{i=1}^N$ denote the dataset, where ${\bf x}_i \in \mathbb {R}^{224 \times 224 \times 3}$ represents the RGB image and $y_i \in \{1, \dots , 10\}$ is the corresponding disease class label. Each image has been processed with normalization and augmentation.

In the normalization step, pixel values are scaled to $[-1, 1]$ using the equation:

1 $$\begin{aligned} \hat{{\bf x}}_i = \frac{{\bf x}_i / 255 - 0.5}{0.5}. \end{aligned}$$

In the data augmentation step for the training phase, we apply domain-informed augmentation techniques that simulate realistic variations in agricultural imaging by random operations (horizontal flip, rotation, width/height shift, zoom, brightness adjustment, and contrast adjustment). We do not apply augmentation operations to validation or test sets to ensure unbiased evaluation. The dataset is divided into 14,616 training images, 1827 validation images, and 1827 test images, with a ratio of 80% : 10% : 10%. The validation set is employed for hyperparameter tuning and early stopping, whereas the test set gives an unbiased assessment of performance.

### Multi-model training

To enable fair comparison, we evaluate seven lightweight architectures in three categories: Standard CNNs (VGG16^34^, ResNet50^35^, DenseNet121^36^); Efficient CNNs (MobileNetV3-Small^8^, ShuffleNetV2^37^, SqueezeNet^10^); and Hybrid CNN-Transformer (MobilePlantViT^16^). Several main parameters of evaluation are listed as follows:

- We use random weight initialisation to ensure fair comparison of models.
- We use Adam^38^ with $\beta _1 = 0.9$, $\beta _2 = 0.999$, and $\varepsilon = 10^{-8}$, Batch size=32.
- We use categorical cross-entropy with L2 weight decay ($\lambda = 10^{-3}$) to prevent overfitting, maximum epochs=200.

### Efficiency assessment

We assess models across three aspects, including diagnostic performance, computational efficiency, and explainability.

#### Diagnostic performance

We evaluate the diagnostic performance of the models through the metrics: overall accuracy, macro-F1 score, class-wise F1 score, and confusion matrix.

#### Computational efficiency

We evaluate the computational efficiency through the following parameters: model size (number of parameters); computational cost (FLOP); and inference latency measured on ONNX Runtime^39^ under two configurations: (a) 4-thread baseline for optimal performance and (b) 1-thread configuration simulating resource-constrained edge devices. These metrics are widely accepted in the literature^8,37^ as reliable samples for the feasibility of edge deployment. However, we acknowledge that actual deployment requires validation on target hardware, including battery consumption, thermal performance, and integration testing, which we leave as future work.

#### Explainability

We use Grad-CAM, LIME, and SHAP with Perturbation Stability Score (PSS) to evaluate all models as detailed in “subsection”.

### Explainable AI (XAI) analysis

For each model, we generate explanations for 50 randomly selected test images (5 per class) using three complementary XAI techniques:

Grad-CAM (gradient-weighted class activation mapping)^23^ is a gradient-based technique that generates visual explanations by calculating the gradient of the predicted class score relative to the feature maps in the final convolutional layer (for CNNs) or the last transformer block (for MobilePlantViT). Formally, the Grad-CAM heatmap $L^c_{\text {Grad-CAM}} \in \mathbb {R}^{H \times W}$ for a class *c* is computed as follows:

2 $$\begin{aligned} L^c_{\text {Grad-CAM}} = \text {ReLU}\left( \sum _k \alpha ^c_k A^k\right) , \end{aligned}$$

where $A^k \in \mathbb {R}^{H \times W}$ is the *k*-th feature map, and $\alpha ^c_k$ is the importance weight:

3 $$\begin{aligned} \alpha ^c_k = \frac{1}{HW} \sum _i \sum _j \frac{\partial y^c}{\partial A^k_{ij}}. \end{aligned}$$

The ReLU activation ensures that only features with a positive influence on the class score are highlighted.

LIME (local interpretable model-agnostic explanations)^40^ is a method that explains predictions by locally approximating the model with an interpretable linear model. LIME produces superpixels using SLIC segmentation, yielding 50 superpixels per image. It then generates 1000 perturbed samples by randomly masking these superpixels and fits a linear model weighted according to proximity to the original instance. The explanation identifies the superpixels that significantly impact the prediction.

SHAP (SHapley Additive exPlanations)^41^ is a game-theoretic approach that provides a Shapley value to each feature (superpixel), reflecting its contribution to the prediction. KernelExplainer is employed with 100 background samples randomly chosen from $\mathscr {D}_{\text {train}}$ to estimate Shapley values. SHAP fulfills key properties, including local accuracy, missingness, and consistency, and ensures theoretically valid feature attributions.

Quantitative robustness evaluation (QRE): following established practices in XAI evaluation ^42,43^, we assess explanation stability using perturbation testing over the metric called *Perturbation Stability Score (PSS)*. For each test image ${\bf x}$, we generate $K=10$ perturbed versions by adding Gaussian noise:

4 $$\begin{aligned} {\bf x}^{(k)} = {\bf x} + \mathscr {N}(0, \sigma ^2 {\bf I}), \quad k=1,\dots ,K, \end{aligned}$$

where $\sigma = 0.01$ represents a small perturbation that does not alter human perception but may affect model predictions. For each perturbed image, we generate a saliency map ${\bf S}^{(k)}$ using the XAI method. The PSS measures the average pairwise similarity between saliency maps:

5 $$\begin{aligned} \text {PSS} = \frac{1}{K(K-1)} \sum _{k=1}^K \sum _{l \ne k} \text {SSIM}({\bf S}^{(k)}, {\bf S}^{(l)}), \end{aligned}$$

where SSIM (structural similarity index)^44^ computes the similarity between two images based on luminance, contrast, and structure. Higher PSS indicates more stable explanations that are robust to small input perturbations, a desirable property for trustworthy AI systems.

## Comparative experimental results

### Experimental setup

All models are trained and evaluated on a workstation equipped with NVIDIA A4000 GPU (16GB VRAM), 48 GB RAM, and Ubuntu 22.04 LTS. We use PyTorch 2.1.0 with CUDA 11.8 for GPU acceleration. The Adam optimizer is configured with an initial learning rate $10^{-4}$ and a weight decay $10^{-3}$. The input resolution is fixed at $224 \times 224$ pixels for all models to ensure fair comparison. Training typically converges within 50-100 epochs for most models, with early stopping preventing overfitting. Due to computational constraints, we report results from a single training run with a fixed random seed for reproducibility, though we acknowledge that multiple runs with different seeds would provide more robust statistics.

### Comparative diagnostic performance

We trained and evaluated each model three times on the PlantVillage dataset using different random seeds. The results, summarized in Table 1, show low variability across runs, indicating good reproducibility and stable training behavior. Our analysis reveals several key findings:

**Table 1:** Diagnostic performance comparison on PlantVillage tomato test set.

Model	Accuracy (mean ± std)	Macro-F1 (mean ± std)
VGG16	$0.9908 \pm 0.0048$	$0.9896 \pm 0.0047$
ResNet50	$0.9948 \pm 0.0018$	$0.9939 \pm 0.0019$
DenseNet121	$0.9961 \pm 0.0013$	$0.9954 \pm 0.0009$
MobilePlantViT	${0.9940 \pm 0.0012}$	${0.9931 \pm 0.0010}$
MobileNetV3-Small	$0.9890 \pm 0.0059$	$0.9887 \pm 0.0061$
SqueezeNetV2	$0.9800 \pm 0.0010$	$0.9753 \pm 0.0016$
ShuffleNetV2	$0.9829 \pm 0.0100$	$0.9770 \pm 0.0128$

DenseNet121 achieves the highest accuracy (99.61%) and macro-F1 (99.31%), followed closely by ResNet50 (99.48%, 99.39%), VGG16 (99.08%, 98.96%), and MobilePlantViT (99.40%, 99.31%). The performance gap between the best and fourth-best models is marginal (<0.2% in accuracy), indicating that several architectures can achieve good classification performance. While DenseNet121 is the best in accuracy, it requires 6.96M parameters and 2.90G FLOPs. Otherwise, MobilePlantViT achieves comparable performance (only 0.21% lower accuracy) with 8.5$\times$ fewer parameters (0.82M) and 4.8$\times$ fewer FLOPs (0.60G), demonstrating superior efficiency. This efficiency advantage translates directly to reduced memory footprint (3.3 MB vs. 27.8 MB for DenseNet121), faster inference, and lower energy consumption.

Among these lightweight models, ShuffleNetV2 achieves the best performance (98.29% accuracy, 97.70% F1) with only 1.26M parameters and 0.15G FLOPs. MobileNetV3-Small, despite being the fastest model (0.06G FLOPs), achieves lower accuracy (98.90%), suggesting that extreme efficiency may sacrifice some diagnostic capability. SqueezeNet, the smallest model (0.73M parameters), achieves the lowest performance (98.00% accuracy), indicating that model capacity is still important for capturing fine-grained disease patterns. VGG16, despite its large size (134.3M parameters), achieves 99.08% accuracy, the same as MobilePlantViT but with 163$\times$ more parameters. This demonstrates the inefficiency of early CNN architectures compared to modern designs. ResNet50 (23.5M parameters) achieves slightly higher accuracy (99.48%) than MobilePlantViT, but with 28$\times$ % more parameters. These results confirm that hybrid CNN-transformer architectures, such as MobilePlantViT, can match the performance of much larger models while maintaining deployment feasibility for edge device scenarios. To improve the practical performance, we enhanced the realism evaluation by conducting robustness tests on the models. We created a robust test dataset by taking data from the PlantVillage test set, then varying the brightness, blurring, noise, and occlusion. We ran the robust test dataset 50 times. In each run, one or more perturbations (brightness adjustment, blurring, noise, or occlusion) were randomly selected and applied to the input images. The results of this robust test are shown in Table 2.

**Table 2:** Robustness evaluation on the PlantVillage dataset.

Model	Accuracy (mean±std)	Macro-F1 (mean±std)
DenseNet121	0.7827 ± 0.0087	0.7846 ± 0.0090
ResNet50	0.7698 ± 0.0102	0.7801 ± 0.0102
VGG16	0.7449 ± 0.0110	0.7592 ± 0.0109
MobilePlantViT	0.7530 ± 0.0104	0.7856 ± 0.0092
MobileNetV3-Small	0.7025 ± 0.0117	0.6855 ± 0.0140
ShuffleNetV2	0.7204 ± 0.0102	0.7426 ± 0.0113
SqueezeNet	0.7085 ± 0.0103	0.7117 ± 0.0112

Table 2 presents the classification performance on the robust test dataset using Accuracy and Macro-F1 (mean ± std). Horizontal rows represent the methods, and columns represent the mean and the standard deviation after 50 tests. The results are quite good at higher difficulty levels because the test images are affected by background noise, lighting changes, blurring, and occlusion of all disease symptoms. MobilePlantViT achieved the highest Macro-F1 and competitive Accuracy, demonstrating balanced and stable classification capabilities across classes. Meanwhile, lighter CNN models such as MobileNetV3-Small, ShuffleNetV2, and SqueezeNet performed worse, showing a trade-off between model lightness and performance in complex field conditions.

### In-field evaluation on PlantDoc (in-domain)

To complement the laboratory-controlled PlantVillage benchmark with an in-field setting, we additionally evaluate the models on the filtered PlantDoc tomato subset (8 classes), which contains natural backgrounds and illumination variations typical of field acquisition. In this setting, we train and evaluate all models *in-domain* on PlantDoc using an 80%/10%/10% train/validation/test split. All results are averaged over three independent runs (different random splits/seeds), and we report mean±std on the PlantDoc test split. To ensure fair comparison across architectures, all models are trained from scratch with random initialization, and no ImageNet pretraining is used.

Table 3 summarizes the results. As expected, PlantDoc performance is consistently lower than the near-saturated PlantVillage results due to real-world imaging conditions. Nevertheless, lightweight models remain competitive on in-field data, supporting their practicality for resource-constrained deployments.

**Table 3:** PlantDoc in-domain results (mean±std over three runs).

Model	Accuracy (mean±std)	Macro-F1 (mean±std)
DenseNet121	0.7684 ± 0.0131	0.7327 ± 0.0199
ResNet50	0.8023 ± 0.0060	0.7717 ± 0.0062
VGG16	0.6690 ± 0.0116	0.5775 ± 0.0248
MobilePlantViT	0.7836 ± 0.0116	0.7327 ± 0.0199
MobileNetV3-Small	0.6830 ± 0.0363	0.6412 ± 0.0343
ShuffleNetV2	0.7860 ± 0.0076	0.7468 ± 0.0078
SqueezeNet	0.5801 ± 0.0066	0.4468 ± 0.0166

### Per-class performance analysis

Table 4 presents per-class F1-scores for all models, revealing performance variations across disease categories. MobilePlantViT achieves perfect F1 scores (1.00) on 6 out of 10 classes (Bacterial Spot, Early Blight, Late Blight, Leaf Mold, Septoria Leaf Spot, and Yellow Leaf Curl Virus). Hence, these results demonstrate strong generalization across diverse disease types with distinct visual characteristics.

**Table 4:** Per-class F1-scores for all models.

Class	DenseNet121	ResNet50	VGG16	MobilePlantViT	MobileNetV3-Small	ShuffleNetV2	SqueezeNet
Bacterial spot	1.00	1.00	1.00	1.00	0.99	1.00	0.99
Early blight	0.99	0.99	0.97	1.00	0.94	0.95	0.94
Late blight	0.99	0.99	0.98	1.00	0.98	0.98	0.97
Leaf Mold	1.00	1.00	1.00	1.00	0.99	0.99	0.97
Septoria spot	1.00	1.00	1.00	1.00	0.97	0.99	0.99
Spider mites	1.00	0.99	1.00	0.99	0.97	0.98	0.98
Target Spot	1.00	0.99	1.00	0.98	0.95	0.97	0.96
Yellow Curl	1.00	1.00	1.00	1.00	1.00	1.00	0.99
Mosaic virus	0.99	0.99	1.00	0.99	0.96	1.00	0.97
Healthy	1.00	1.00	1.00	1.00	1.00	1.00	0.99

### Confusion matrix analysis

**Fig. 2:**
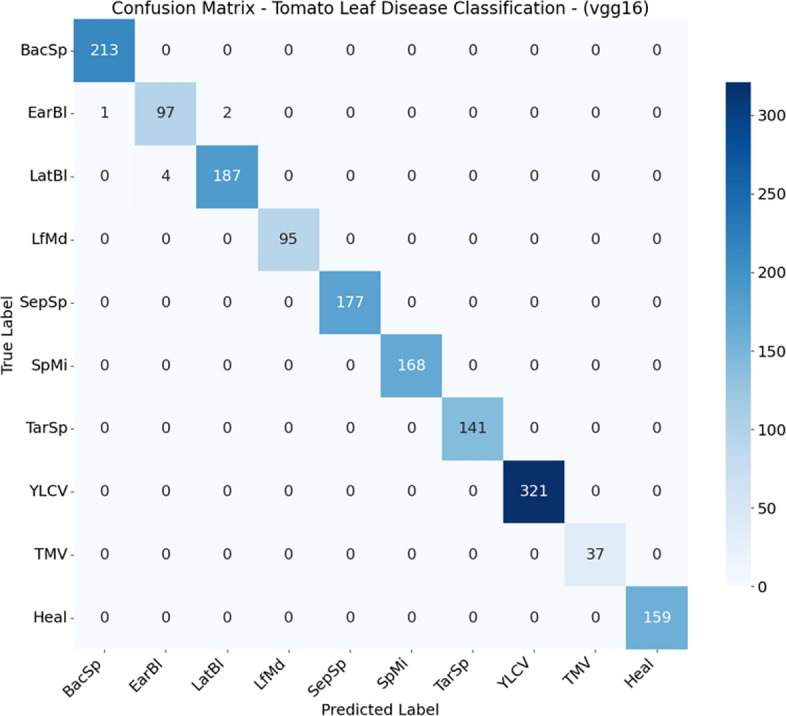
Confusion matrix for VGG16 on the test set.

**Fig. 3:**
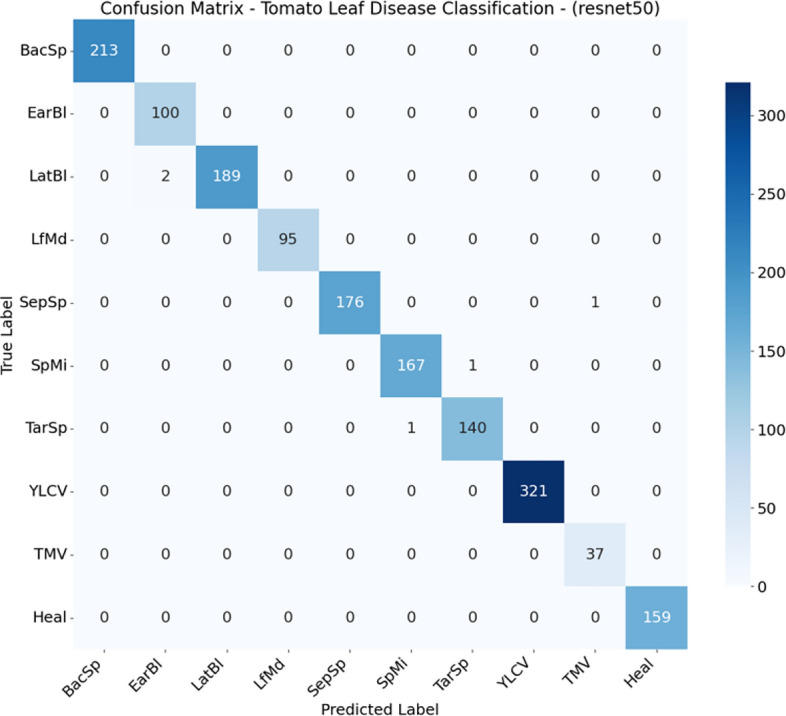
Confusion matrix for ResNet50 on the test set.

**Fig. 4:**
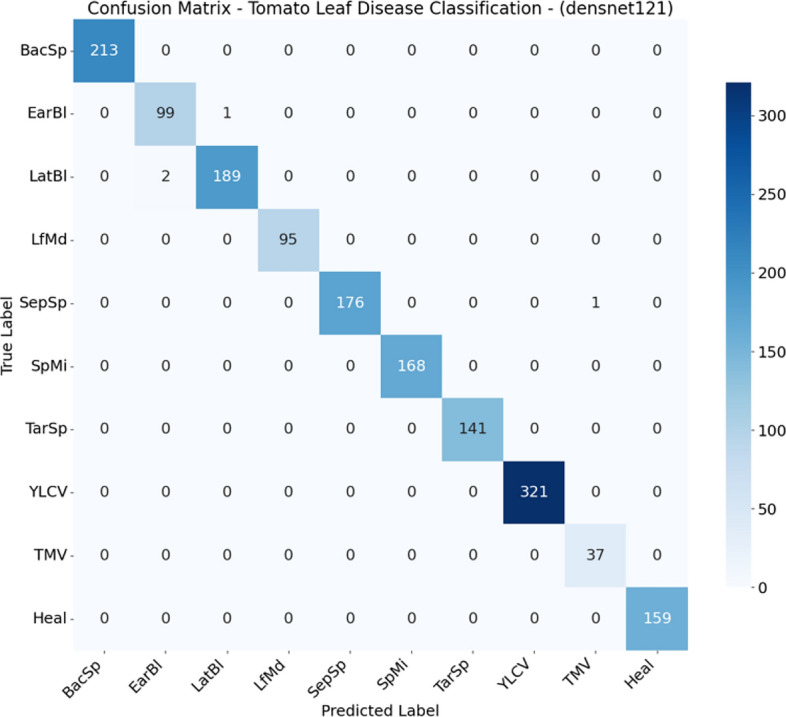
Confusion matrix for DenseNet121 on the test set.

**Fig. 5:**
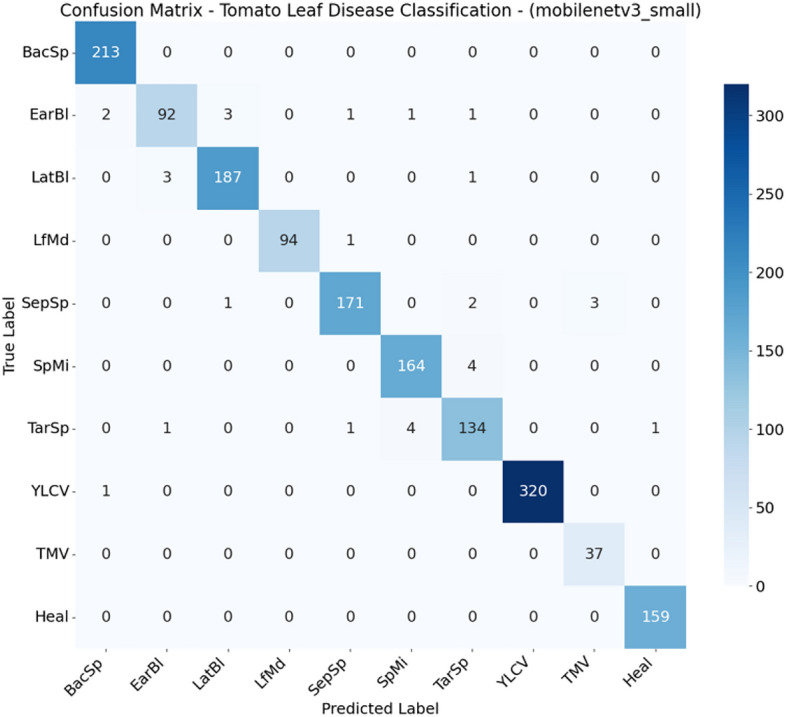
Confusion matrix for MobileNetV3-small on the test set.

**Fig. 6:**
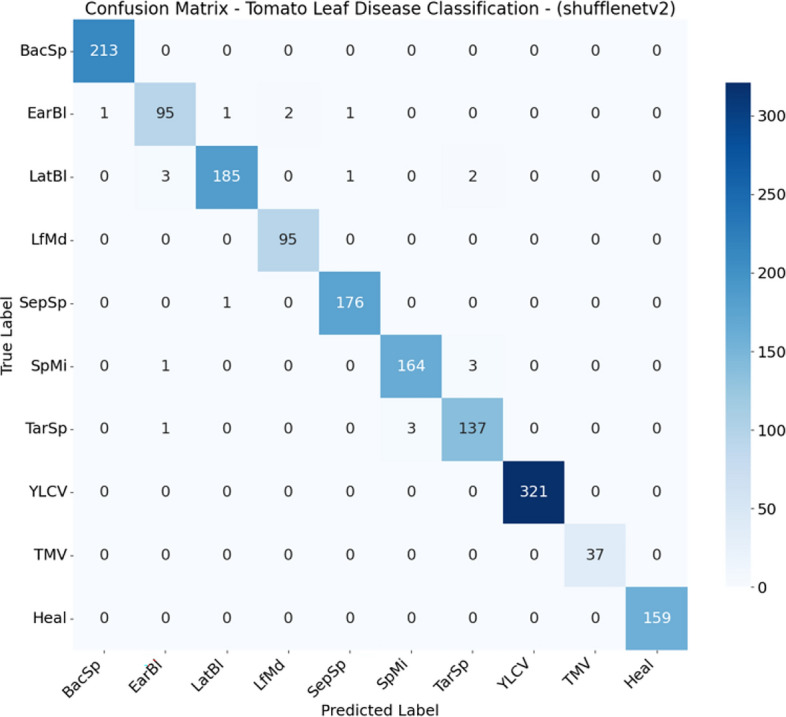
Confusion matrix for ShuffeNet on the test set.

**Fig. 7:**
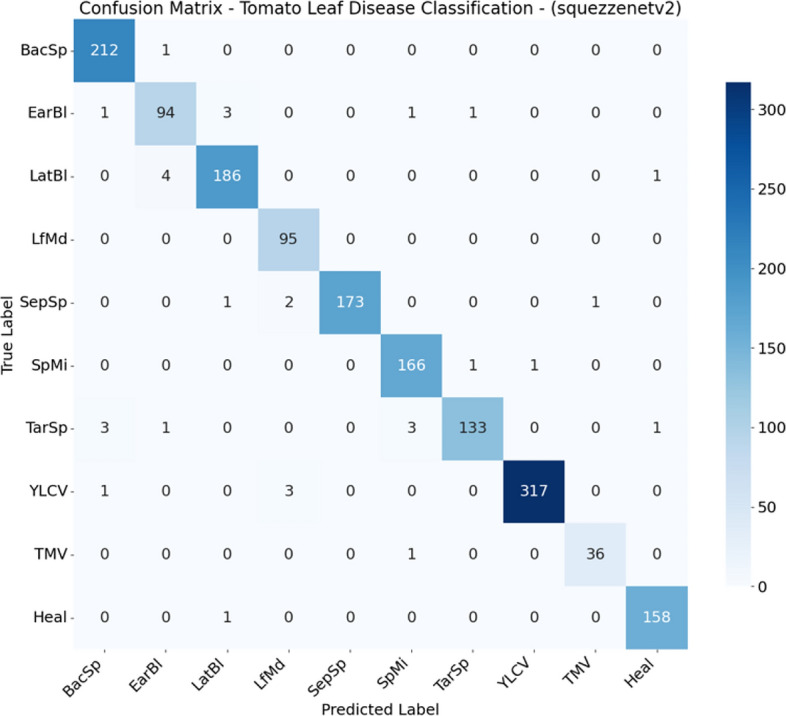
Confusion matrix for SqueezeNet on the test set.

**Fig. 8:**
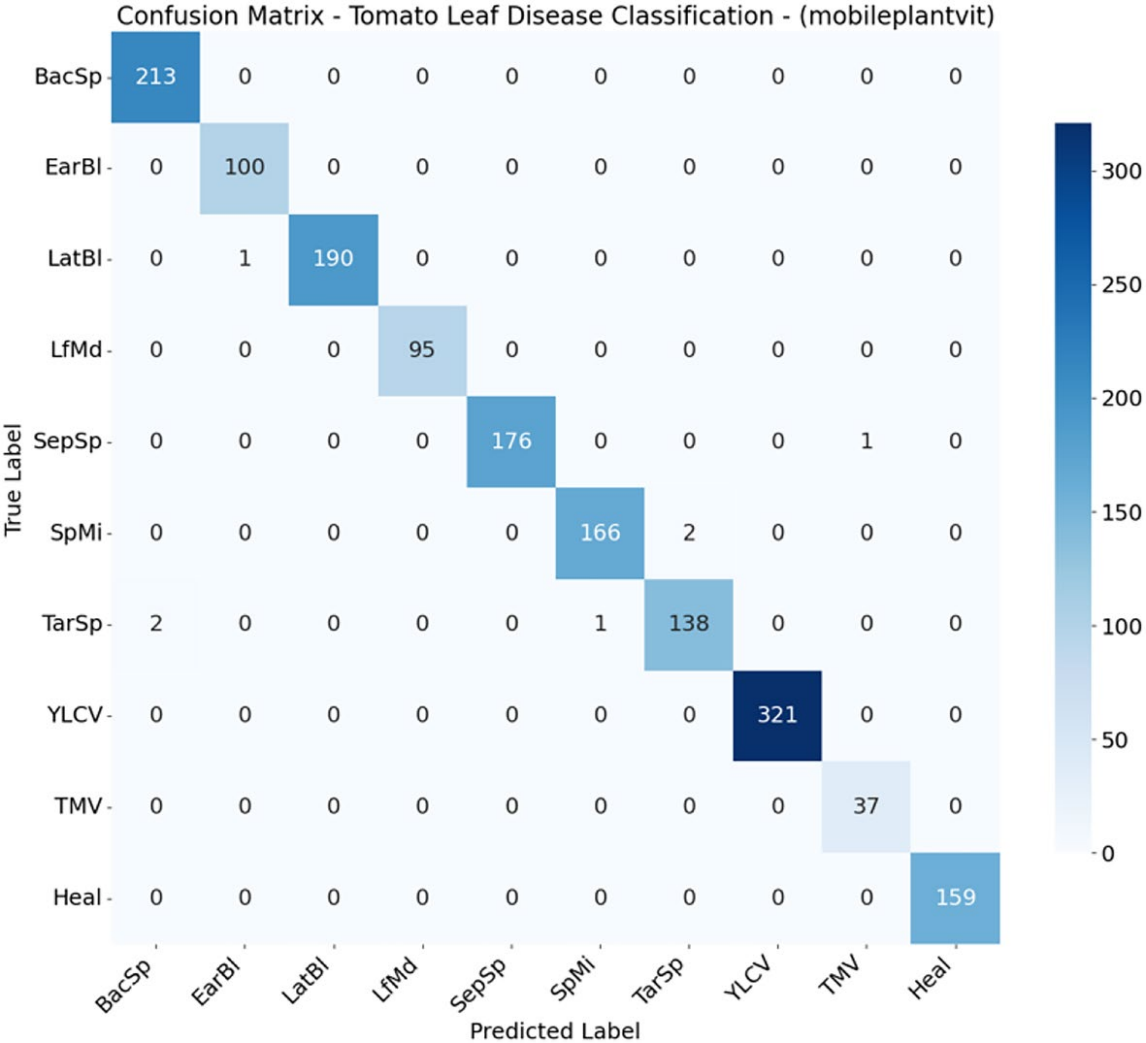
Confusion matrix for MobilePlantViT on the test set.

From Figs. 2, 3, 4, 5, 6, 7, and  8 present the confusion matrices for the applied models. To help clear text in the figures, the following abbreviations are used to denote tomato leaf conditions: BacSp (Bacterial Spot), EarBl (Early Blight), LatBl (Late Blight), LfMd (Leaf Mold), SepSp (Septoria Leaf Spot), SpMi (Spider Mites), TarSp (Target Spot), YLCV (Yellow Leaf Curl Virus), TMV (Tomato Mosaic Virus), and Heal (Healthy Leaf). We can see that the efficient and hybrid CNN model group, such as MobilePlantViT, DenseNet121, and ResNet50, achieve near-perfect classification performance on the testing dataset.

Although the models distinguish well between healthy and diseased leaves, there have been reports of confusion between early blight and late blight, which have similar symptoms (brown spots), and Target Spot and Septoria Leaf Spot, which have the same small circular shape.

### Computational efficiency analysis

Table 5 presents model complexity metrics. MobilePlantViT achieves an optimal balance with only 0.82M parameters (the smallest, except for SqueezeNet) and 0.60G FLOPs, significantly lower than standard CNNs while maintaining competitive accuracy.

**Table 5:** Model complexity and computational cost.

Model	Params (M)	FLOPs (G)
DenseNet121	6.96	2.90
ResNet50	23.53	4.13
VGG16	134.30	15.47
MobilePlantViT	0.82	0.60
MobileNetV3-Small	1.53	0.06
ShuffleNetV2	1.26	0.15
SqueezeNet	0.73	0.26

Table 6 presents inference latency measurements on ONNX Runtime (CPU-only mode, Intel i7-12700) under two configurations: 4-thread (baseline) and 1-thread (resource-constrained). ONNX Runtime is configured with 2 threads, with built-in graph optimizations (e.g., operator fusion) enabled. The inference latency is measured by averaging the results of 100 runs on a single input image. A warm-up phase is performed before measurement to ensure stability. The reported latency excludes both pre-processing and post-processing times. MobilePlantViT achieves 21.30 ms per 1 images on a 4-thread configuration and 69.11 ms per 1 images on a 1-thread configuration.

**Table 6:** Inference latency on ONNX runtime (CPU-only, Intel i7-12700).

Model	1-thread (ms/img)	4-thread (ms/img)
DenseNet121	224.26	75.33
ResNet50	396.63	121.30
VGG16	12509.61	3894.99
MobilePlantViT	69.11	21.30
MobileNetV3-Small	16.24	6.50
ShuffleNetV2	8.21	3.21
SqueezeNet	52.29	20.63

Edge deployment feasibility: the 4-thread configuration represents optimal performance when sufficient CPU cores are available, achieving an inference latency of 21.30 ms per image ($\approx$47 FPS), which satisfies real-time requirements (>30 FPS). The 1-thread configuration simulates resource-constrained edge devices where only a single CPU core is allocated to inference, as commonly observed in IoT and low-power embedded systems. Under this constraint, MobilePlantViT maintains a latency of 69.11 ms per image ($\approx$14.5 FPS), which remains suitable for near real-time agricultural monitoring tasks where response intervals of 100–500 ms are typically acceptable for handheld or field scanning devices. The 3.2$\times$ increase in latency from 4 threads to 1 thread is mainly attributed to reduced parallelism in convolutional and transformer attention operations when multithreading is not available. Furthermore, MobilePlantViT requires approximately 3.3 MB in FP32 format (0.82 M parameters $\times$ 4 bytes), which fits comfortably within the memory capacity of typical edge AI platforms such as Raspberry Pi–class devices. In addition, the model exhibits low computational complexity (0.60 GFLOPs), which is generally associated with lower energy consumption on resource-constrained hardware. As shown in Table 6, MobilePlantViT demonstrates substantial multi-threading acceleration (3.24$\times$ speedup), trailing only ResNet50 (3.27$\times$), which confirms that its hybrid CNN-Transformer design efficiently leverages parallel computation. These results establish MobilePlantViT as the optimal architecture for applications requiring both high diagnostic accuracy (>99.5%) and deployment feasibility on resource-constrained edge devices.

### Edge deployment on Raspberry Pi 5

To strengthen the edge deployment claim beyond desktop CPU estimates, we benchmark all exported ONNX models on a Raspberry Pi 5 in CPU-only mode using ONNX Runtime. We report end-to-end inference latency (ms/image), throughput (FPS), peak RAM usage, and CPU utilization under two configurations: *1-thread* (constrained) and *4-thread* (parallel). The ONNX Runtime configuration is set up to be the same as the desktop configuration. All measurements use batch size 1 and input resolution $224 \times 224$. To reduce measurement noise, we perform a warm-up phase before timing and then average latency and throughput over a fixed number of images. Table 7 summarizes the on-device benchmark results.

**Table 7:** On-device inference benchmark on Raspberry Pi 5 (CPU-only) using ONNX runtime.

Model	Threads	Latency (ms/img)	FPS	Peak RAM (MB)	CPU usage (%)
DenseNet121	1	348.15	2.87	136.1	39.8
ResNet50	1	419.96	2.38	202.0	26.2
VGG16	1	1446.85	0.69	648.2	26.1
MobilePlantViT	1	252.26	3.96	131.0	26.4
MobileNetV3-Small	1	19.71	50.73	99.1	26.3
ShuffleNetV2	1	22.19	45.06	112.3	26.3
SqueezeNet	1	32.85	30.44	112.3	26.3
DenseNet121	4	131.64	7.60	137.4	80.3
ResNet50	4	154.84	6.46	202.7	79.6
VGG16	4	518.60	1.93	662.4	80.1
MobilePlantViT	4	101.46	9.86	130.1	80.3
MobileNetV3-Small	4	9.00	111.06	99.1	79.5
ShuffleNetV2	4	9.98	100.15	112.3	80.0
SqueezeNet	4	14.76	67.73	112.3	80.5

As shown in Table 7, the Raspberry Pi results confirm that MobilePlantViT can be executed on a low-power edge CPU with a modest memory footprint (about 130 MB peak RAM). While MobilePlantViT does not target video-rate processing on CPU-only Raspberry Pi, it achieves stable throughput close to 10 FPS under 4-thread execution, which is suitable for interactive or periodic capture diagnosis scenarios in practical agricultural deployments.

### Explainability analysis

**Fig. 9:**
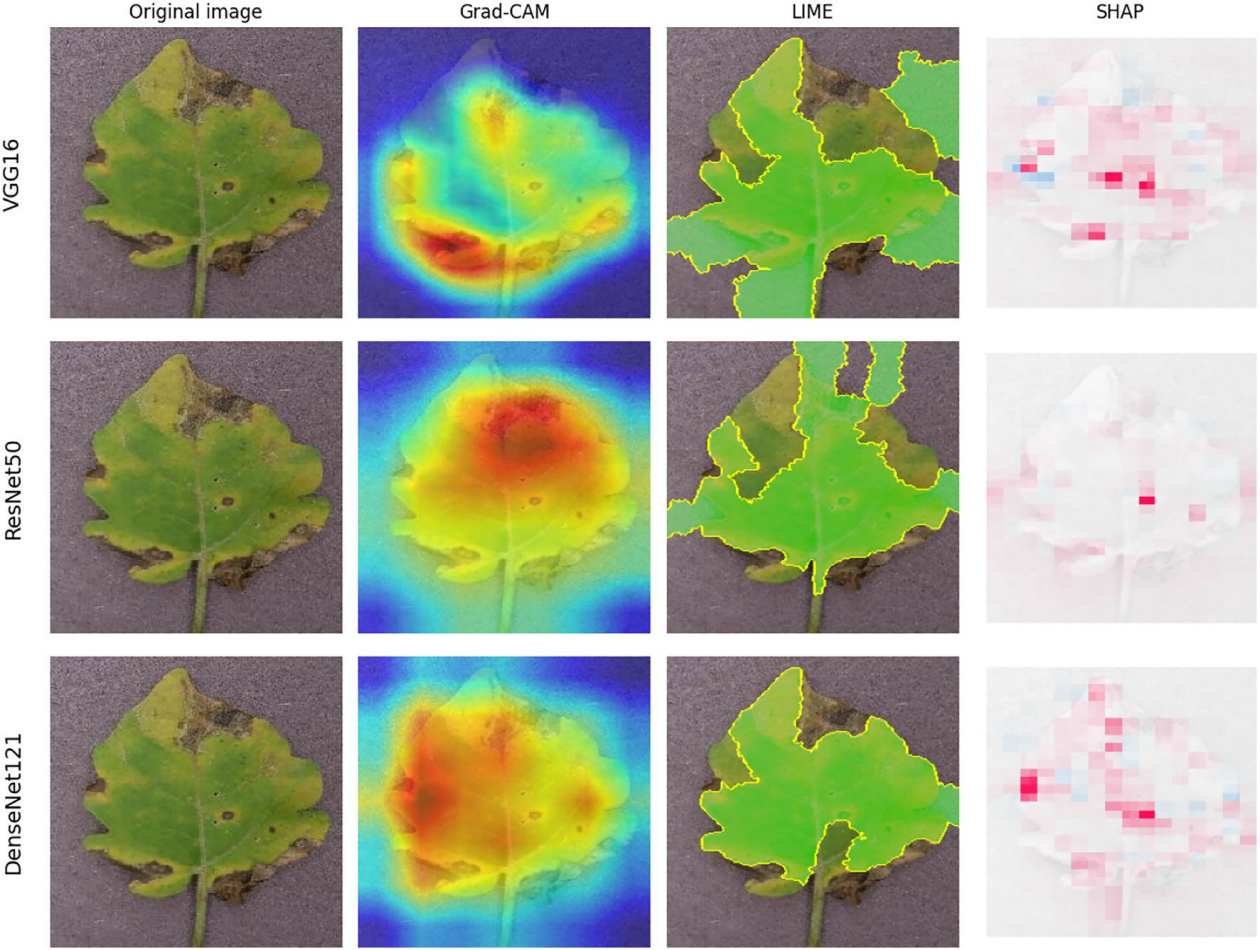
XAI visualizations standard CNNs architectures.

Figure 9 illustrates the explanation results for the Standard CNNs. All three models produce high-quality and similar Grad-CAM explanations, which reflect their near-perfect classification performance. Similarly, LIME and SHAP also identify the superpoints that contribute significantly to the prediction. However, the spatial resolution is lower than Grad-CAM. Hence, the standard CNN model produces reliable and visually robust explanations.

**Fig. 10:**
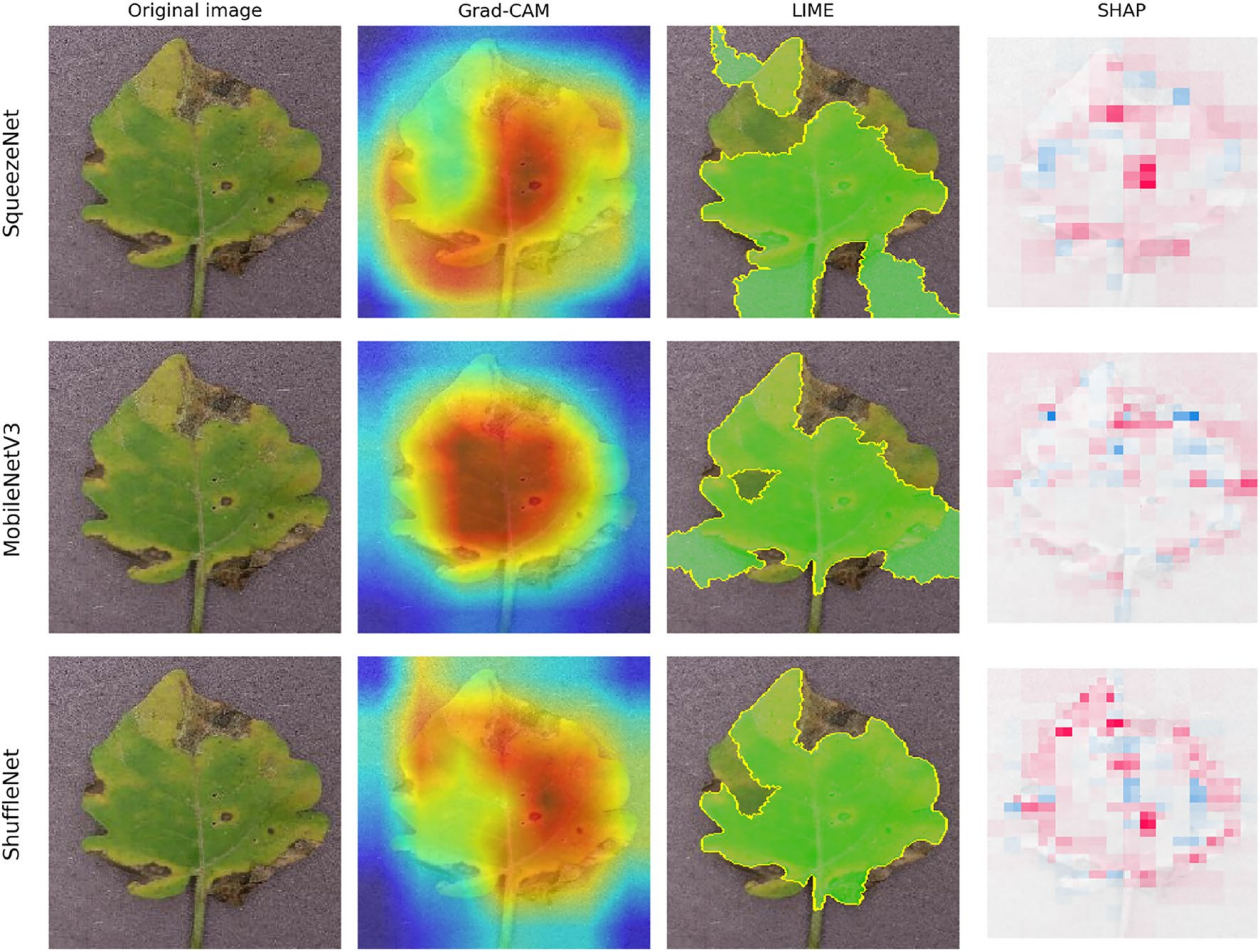
XAI visualizations efficiency CNNs architectures.

Figure 10 illustrates the explanation results for the efficient CNNs. Efficient CNNs have shown impressive performance. Although the heatmaps from the efficient models may be slightly blurry or spread out, they are still sufficient to indicate the exact area of the symptom. When compared to Table 8, the high scores indicate that the explanations of these models are stable against input noise.

**Fig. 11:**
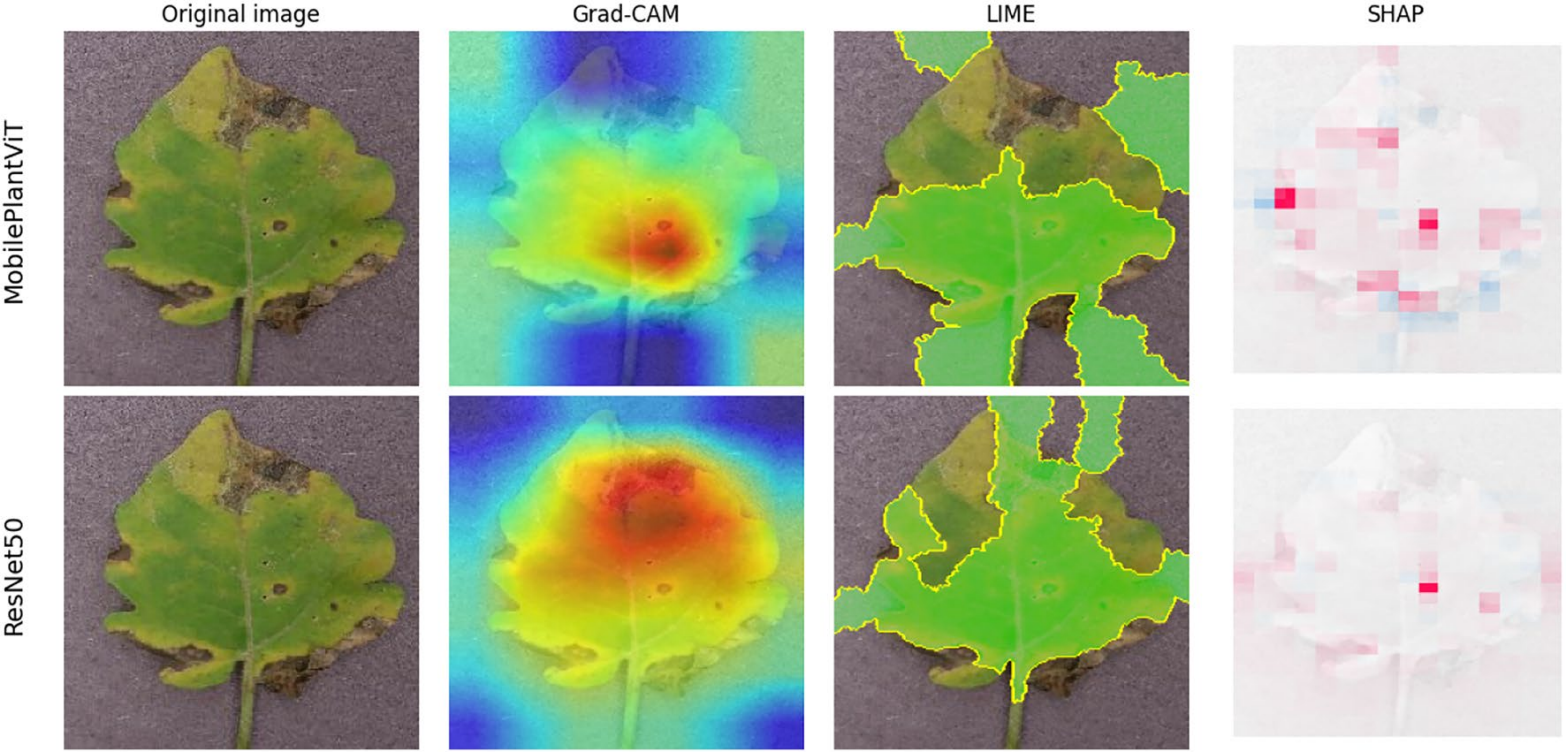
XAI visualizations of MobilePlanVit vs Restnet architectures.

Figure 11 visually compares the model explanation quality (XAI) between MobilePlantViT and ResNet50 (which is the most effective model in the set of effective CNN models). The heatmap results show that MobilePlantViT still accurately and clearly locates diseased areas (such as brown spots and yellow leaves), similar to ResNet50, although with much fewer parameters. Table 8 presents the average Perturbation Stability Score (PSS) scores across 50 test images using the three proposed XAI methods. The horizontal rows represent the models under investigation. The vertical columns display the PSS of the XAI method in comparison to the original image at various noise levels. Each test image was Gaussian-noised at levels of 0.01, 0.03, 0.05, 0.07, 0.09, and 0.1. Table  8 shows that all models achieved high and very high stability with low noise levels, and this stability decreased as the noise level increased.

**Table 8:** PSS scores under different noise levels.

Model	$\sigma$					
	0.01	0.03	0.05	0.07	0.09	0.10
**Grad-CAM**						
DenseNet121	0.9703	0.8226	0.6518	0.5041	0.3916	0.3423
ResNet50	0.9675	0.8124	0.6322	0.4797	0.3699	0.3263
VGG16	0.9615	0.7965	0.6191	0.4782	0.3724	0.3339
MobilePlantViT	0.9401	0.7319	0.5349	0.4002	0.3209	0.2892
MobileNetV3-Small	0.9628	0.8105	0.6294	0.4839	0.3811	0.3390
ShuffleNetV2	0.9466	0.7706	0.5835	0.4394	0.3472	0.3109
SqueezeNet	0.9642	0.8095	0.6392	0.4970	0.3909	0.3496
**LIME**						
DenseNet121	0.8269	0.6282	0.4595	0.3440	0.2627	0.2344
ResNet50	0.8245	0.6297	0.4576	0.3384	0.2614	0.2323
VGG16	0.8256	0.6312	0.4611	0.3455	0.2669	0.2372
MobilePlantViT	0.8306	0.6294	0.4580	0.3408	0.2595	0.2292
MobileNetV3-Small	0.8176	0.6296	0.4638	0.3472	0.2658	0.2354
ShuffleNetV2	0.8175	0.6258	0.4627	0.3461	0.2670	0.2350
SqueezeNet	0.8252	0.6358	0.4697	0.3527	0.2710	0.2385
**SHAP**						
DenseNet121	0.9741	0.9252	0.8770	0.8381	0.8055	0.7927
ResNet50	0.9777	0.9288	0.8849	0.8452	0.8068	0.7933
VGG16	0.9753	0.9231	0.8777	0.8459	0.8092	0.8033
MobilePlantViT	0.9594	0.9004	0.8446	0.8050	0.7837	0.7718
MobileNetV3-Small	0.9691	0.9161	0.8693	0.8151	0.7890	0.7777
ShuffleNetV2	0.9655	0.9101	0.8642	0.8169	0.7920	0.7755
SqueezeNet	0.9723	0.9212	0.8792	0.8357	0.8079	0.7976

**Fig. 12:**
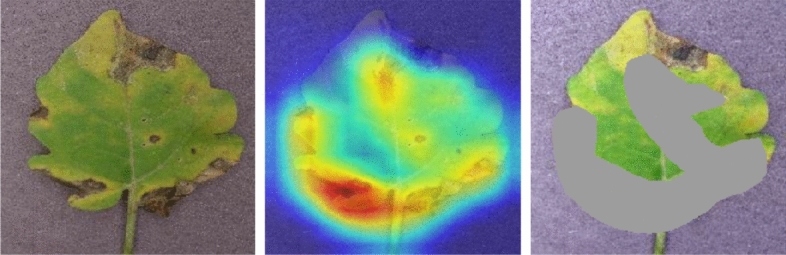
Visualization of Grad-CAM heatmap and masked input after removing salient regions.

Of the three interpretation methods, SHAP consistently achieved the highest PSS values and showed the slowest degradation as noise levels increased, indicating strong stability under perturbation. Grad-CAM exhibited moderate stability, with PSS decreasing more significantly at higher noise levels, while LIME produced the lowest PSS values, suggesting that the local alternative interpretations are more sensitive to input noise. When comparing the model architectures, MobilePlantViT maintained relatively high and stable PSS across all methods, indicating that its interpretations are less affected by perturbation and remain consistent even under degraded input conditions, while some conventional CNN models, such as VGG16 and DenseNet121, showed more pronounced degradation at higher noise levels. These results demonstrate that PSS effectively captures the stability of interpretation, and consistent trends across different XAI methods support the reliability of this index; in practical agricultural diagnostics on edge devices, where images may contain sensor noise or light variations, such stability is essential to ensure reliable and interpretable predictions. Fig. 12 illustrates the faithfulness test technique by covering 30% of the most prominent region, as indicated by the Grad-CAM heat map, to assess the faithfulness of the explanation. The results show that when covering the important regions explained by Grad-CAM, all models experienced a significant drop in prediction confidence. This proves that the explanation of the Grad-CAM method is completely accurate. The results are shown in Table  9.

**Table 9:** Confidence degradation after Grad-CAM–guided salient region masking.

Model architecture	Original confidence	Masked confidence	Confidence drop
DenseNet121	0.9988	0.0006	0.9982
ResNet50	0.9996	0.0009	0.9988
VGG16	0.9999	0.0201	0.9798
MobilePlantViT	0.9992	0.0034	0.9958
MobileNetV3	0.9996	0.0001	0.9995
ShuffleNet	0.9996	0.0029	0.9967
SqueezeNet	0.9464	0.0035	0.9429

Table 9 assesses the accuracy of Grad-CAM interpretations through a masking experiment, in which the most prominent regions were masked while all other conditions remained unchanged. The consistently large confidence drop ($\ge 0.94$) across all models suggests that the highlighted regions contain features that are causally important for classification, as their removal causes predictions to collapse. MobilePlantViT showed both high initial confidence and a large drop in confidence, indicating that its decisions depend heavily on disease-related regions. The slightly lower drop in VGG16 and SqueezeNet implies more dispersed attention. Because the masking procedure was applied uniformly and exhibited a direct causal interference, the results provide strong quantitative evidence that the interpretations are accurate and meaningful for plant disease diagnosis.

## Limitations and future work

Despite promising results, several limitations should be noted. First, the evaluation was conducted on the PlantVillage dataset, which contains laboratory-controlled images; further validation on field-acquired datasets is needed to assess real-world robustness. Second, latency measurements were obtained from CPU-based inference experiments and have not yet been validated across diverse physical edge devices. Third, while the PSS metric evaluates explanation stability, it does not measure fidelity to ground-truth disease regions, which should be investigated in future studies. Future work will therefore focus on validation under real field conditions, incorporation of fidelity-based explainability metrics, and further optimization for deployment on practical edge computing platforms.

## Conclusion

This paper presented a systematic comparison of seven lightweight deep learning architectures for tomato disease classification, evaluating their diagnostic accuracy, computational efficiency, and explainability with a focus on edge deployment. The experimental results demonstrate that MobilePlantViT provides the best overall balance between accuracy and efficiency, achieving 99.40% accuracy with significantly fewer parameters and lower computational cost than larger CNN-based models, while maintaining real-time CPU inference performance under the tested configuration. In addition, quantitative explainability analysis indicates that Grad-CAM produces consistently stable visual explanations across different architectures. Overall, these findings suggest that lightweight hybrid architectures combining convolutional and transformer components represent a promising direction for practical plant disease diagnosis systems, particularly in resource-constrained environments.

## Data Availability

The datasets generated and/or analysed during the current study were obtained from two publicly available datasets (PlantVillage and PlantDoc) to create subsets of tomato data. We enhanced the quality of the PlantDoc dataset by collaborating with experts to identify and crop regions containing disease-specific symptoms, while eliminating irrelevant image content. For long-term preservation and ease of access, we have stored copies of the datasets in the published repository. The datasets are available at the following links: https://www.kaggle.com/datasets/cthngon/tomato-plantvillage-datasets, https://www.kaggle.com/datasets/cthngon/tomato-only.
